# Supplementary material for: Mercury Methylation Potentials in Sediments of an Ancient Cypress Wetland Using Species-Specific Isotope Dilution GC-ICP-MS
Source: Molecules. 2022 Aug 1;27(15):4911. doi: 10.3390/molecules27154911 (PMC9370401; doi:10.3390/molecules27154911)
Supplement: Supplementary file 1 [file molecules-27-04911-s001.zip › molecules-1833614-supplementary.pdf]

Supplementary Material

Detailed Description of the Method of Extracting and Isolating MeHg from Sediments:

1. A distillation tube was placed on an analytical balance and the balance was tared.

2. About 0.5 g of sediment was transferred and weighed.

3. About 0.05 g of the enriched spike of $CH_3{}^{201}Hg^+$ (29.02 ng/g) was added to each sample. It is important to weigh the spike as this weight is necessary for the isotope dilution equation.

4. 25 g of Ultrapure water (>18 MΩ cm^{-1}), 0.5 mL of 20% KCl, and 1 ml of 50% H_2SO_4 was then added to the distillation vials.

5. About 5 g of Ultrapure water (>18 MΩ cm^{-1}) was added to the 30 mL receiving vials, the vials were then placed in ice.

6. The N_2(g) gas flow was set to 80 mL/min for each flow meter. Each vessel had N_2(g) bubbling connected through them. The hot-block temperature was set to 120°C.

7. The distillation took between 4-6 hours for completion. The receiving vessels were capped when they contain about 20-25 ml of solution.

8. About 0.5 g of the distillate was transferred into a brown-amber vial. Next 225 μL of acetate buffer was pipetted, and topped off with Deionized H_2O to about 30 g.

9. The final step was the most time sensitive step. The addition of $NaBEt_4$ was used as ethylating agent and as this is a highly volatile compound, care must be done to quickly complete this

step. $NaBEt_4$ was taken out from the freezer, warmed up and then quickly 30 μL were pipetted into each sample container. After the $NaBEt_4$ solution was pipetted, the samples were capped, and were analyzed immediately by the GC-ICP-MS.
